# Improved Stability of a Model IgG3 by DoE-Based Evaluation of Buffer Formulations

**DOI:** 10.1155/2016/2074149

**Published:** 2016-03-03

**Authors:** Brittany K. Chavez, Cyrus D. Agarabi, Erik K. Read, Michael T. Boyne II, Mansoor A. Khan, Kurt A. Brorson

**Affiliations:** ^1^Division II, Office of Biotechnology Products, OPQ, CDER, FDA, Silver Spring, MD 20903, USA; ^2^Division of Product Quality Research, Office of Testing and Research, OPQ, CDER, FDA, Silver Spring, MD 20903, USA; ^3^Division of Pharmaceutical Analysis, Office of Testing and Research, OPQ, CDER, FDA, Silver Spring, MD 20903, USA

## Abstract

Formulating appropriate storage conditions for biopharmaceutical proteins is essential for ensuring their stability and thereby their purity, potency, and safety over their shelf-life. Using a model murine IgG3 produced in a bioreactor system, multiple formulation compositions were systematically explored in a DoE design to optimize the stability of a challenging antibody formulation worst case. The stability of the antibody in each buffer formulation was assessed by UV/VIS absorbance at 280 nm and 410 nm and size exclusion high performance liquid chromatography (SEC) to determine overall solubility, opalescence, and aggregate formation, respectively. Upon preliminary testing, acetate was eliminated as a potential storage buffer due to significant visible precipitate formation. An additional 2^4^ full factorial DoE was performed that combined the stabilizing effect of arginine with the buffering capacity of histidine. From this final DoE, an optimized formulation of 200 mM arginine, 50 mM histidine, and 100 mM NaCl at a pH of 6.5 was identified to substantially improve stability under long-term storage conditions and after multiple freeze/thaw cycles. Thus, our data highlights the power of DoE based formulation screening approaches even for challenging monoclonal antibody molecules.

## 1. Introduction

The manufacturing of biotechnology products is a complex logistical process that connects multiple unit operations and often leads to lengthy in-process hold times or bulk drug substance storage. Identification of appropriate storage conditions and optimized buffer systems for biopharmaceutical proteins is essential in ensuring the stability of these products and therefore maintaining the purity, potency, safety, and efficacy of these drug substances throughout the manufacturing process. A typical purification scheme for monoclonal antibodies involves Protein A affinity chromatography followed by polishing chromatography and filtration steps, with an end product of concentrated antibody in a mild acid to neutral pH solution, prior to drug substance formulation. Selection of a suitable buffer system that mitigates physical and chemical degredation of monoclonal antibodies, especially one that minimizes aggregate and particle formation is an important consideration for efficient downstream fill-finish operations and long-term stability [1]. Parameters that are typically studied include solution pH, buffering system, inclusion of saccharides, tonicity agents, detergents, and other excipients [2, 3].

Regulatory guidance stipulates that antibodies intended for human subjects are tested both at lot release and in stability studies [4] for a variety of product attributes, including opalescence and degradation products such as aggregates, particles, or precipitate formation. These undesirable degradation products may be associated with immune responses [5] and in extreme cases can lead to loss of significant monomer content or protein insolubility, impacting potency, and efficacy to the point where it is unacceptable to use in humans.

In this study we use a monoclonal antibody cell culture system that was developed by hybridoma technology and has been used by several academic groups to evaluate different aspects of manufacturing from cell culture to formulated bulk drug substance [6–10]. This model murine IgG3, while not a humanized antibody suitable for clinical use, has no proprietary entanglements and can be successfully used as a model for bioreactor produced monoclonal antibodies. Its production system was previously adapted to serum-free suspension bioreactor culture and used by several groups to evaluate cell culture bioprocesses both in single run experiments and in design of experiment (DoE) formats [11–13]. We have subsequently found that certain aspects of its biochemistry present a stringent challenge model for formulation development. Acetate buffer can be used for other antibodies [2], but it seems to cause aggregation and precipitation in the case where it is difficult to formulate model antibody.

Prior experience with this antibody (data not shown) showed that it formed visible particulates over time at concentrations above 5 mg/mL to the extent of noticeable loss of monomeric species over time. The aggregation was further exacerbated by freeze/thaw cycles (data not shown). While this drug substance model antibody has been stable enough for short-term storage in 50 mM arginine and 100 mM NaCl, pH 8.0 prior to use in drug product lyophilization studies [14], a stable model antibody solution is needed for long-term quality assessment and testing. In addition, by performing this exercise with our model antibody, we present a rigorous test case for demonstrating the power of DoE approaches for liquid antibody formulation development.

To this end, we demonstrated the power of DoE based studies to quickly pinpoint suitable buffer formulations to maximize the stability of this antibody. We tested four different buffer systems that were chosen to possess a range of pH optima while also avoiding the antibody's known isoelectric point (pI) range 8.4–8.8. The DoE approach enables comprehensive evaluations of relevant formulation parameters that can impact antibody stability.

## 2. Materials and Methods

### 2.1. Reagents

Buffers were prepared using components commonly employed to formulate antibodies: L-Histidine (Sigma-Aldrich, St. Louis, MO), Sodium Chloride (BDH, Radnor, PA), Hydrochloric Acid (Fisher, Fairlawn, NJ), and either L(+)-Arginine (Acros Organics, Waltham, MA) or Freebase Arginine (Fisher). NuPAGE LDS Sample Buffer, NuPAGE Reducing Agent, NuPAGE Antioxidant and Novex Sharp Standard, and MOPS were obtained from Invitrogen (Carlsbad, CA). Brilliant Blue G-250, acetic acid, and 2-propanol were obtained from Fisher Scientific. Unless noted otherwise in the text, reagents were as described in Read et al. [7].

### 2.2. IgG Production

A suspension adapted murine hybridoma that produces IgG3/*κ* antibody [15] was grown in a 7.5-liter Bioflo 110 bioreactor (New Brunswick Scientific, Edison, NJ) that contained 4 liters of media as described in Read et al. [7]. Antibodies from the clarified cell culture fluid (CCF) were captured with a 25 mL Prosep A (Millipore, Billerica MA) column run on an AKTA Avant (GE Healthcare, Uppsala, Sweden) and eluted with 1 M Arginine pH 4.0 [16]. As described in other studies, this elution strategy results in two peaks, an early peak containing mostly host cell proteins, and a subsequent peak containing largely intact antibody [13, 16]. Fractions that comprise the second elution peak were then tested by UV to confirm protein content prior to pooling, buffer exchange, and analytical methods described below.

### 2.3. Preliminary Experimental Design

An initial exploration of three common buffer systems was performed by a 2^3^ full factorial DoE with a center point (Table 1). Experience with the IgG3 antibody used in this study revealed that it was a challenging model from the standpoint of stability and propensity to precipitate (data not shown). Early attempts to find a suitable single species buffer system (including phosphate, tris, acetate, histidine, and citrate) encompassing a range of mildly acidic or neutral pH failed to produce a system where opalescence, or even gross precipitation, did not accumulate over time. Given the need to establish a suitable buffer system for this model antibody, we initiated a controlled evaluation of commonly used single species buffer systems (acetate, histidine, and arginine) described in Table 1. While arginine has limited buffering capacity in the neutral pH range, it was chosen as a mild chaotropic agent that has been reported to stabilize antibodies prone to aggregation [16]. The following full factorial DoEs evaluated each buffer species while varying NaCl, pH away from the antibody isoelectric point, and buffer species concentration. The statistical design, experimental randomization, and analysis were performed on JMP version 10.0 (SAS Institute Inc., Cary, NC).

### 2.4. Sample Analysis Plan

To buffer exchange the 1 M arginine stabilized antibody into the test single buffer species formulation buffers, a 3 mL aliquot of IgG3 at 2 mg/mL or above was loaded into a 10 kDa molecular weight cutoff Slide-A-Lyzer cassette (Thermo Scientific, Rockford, IL). It was dialyzed in the test formulation buffer overnight, equivalent to an 18,000-fold buffer exchange. Dialyzed samples were collected, weighed to determine postdialysis volume, and visually inspected for the presence of gross precipitate and opalescence. To monitor long-term stability, SEC, Protein A, and absorbance measurements at 280 nm (protein content) and 410 nm (opalescence) were performed at day 0 (*T*0), 30 days in 4°C (*T*30), and after three cycles of freezing (−80°C held for 2 hours) and thawing (F/T) (37°C for 10 minutes) for the arginine and histidine buffer formulations. The remaining 9 acetate formulations were not fully tested based on initial analytics indicating decreased stability of the antibody at* T*0.

### 2.5. Experimentally Derived 2^4^ Full Factorial DoE

A 2^4^ full factorial combined arginine and histidine systems for an additional 16 buffer formulations. Test articles from the His/Arg (HR) DoE were analyzed by the same procedures described in Table 2.

### 2.6. UV/VIS (A280 nm/A410 nm) Analyses

A NanoDrop 2000c system was blanked with the test buffer before measuring absorbance of the samples at 280 nm and 410 nm. Samples were not centrifuged before these readings so as not to skew the 410 nm absorbance which accounts for opalescence/visible particulates. To make sure that the 280 nm measurement was within the instrument linear range, samples were then diluted 10-fold and reanalyzed. Any samples that showed an* A*410 reading of 0.2 or greater were considered poor candidates for further optimization, and further analytics were discontinued.

### 2.7. SEC

Analytical scale size exclusion chromatography (SEC) was performed with a TSKgel G3000SWxl column (Tosoh Bioscience, Grove City, OH) and Agilent 1200 HPLC system. These data were used to determine the relative proportion of aggregates with the antibody samples [7]. Visible particulates were removed by centrifugation prior to HPLC analysis to prevent clogging of the frit.

### 2.8. SDS-Page Gel (Reduced and Nonreduced)

Samples (200 *μ*L) were centrifuged at 17,000 ×g to create soluble (supernatant) and insoluble (pellet) fractions. The supernatant was recovered directly for analysis. The pellet was washed with the corresponding test buffer formulation before it was resuspended in 20 *μ*L of sterile water. Both fractions were mixed 1 : 1 with loading buffer (containing DTT for reduced samples) and held at 70°C in a water bath for 10 minutes. 15 *μ*L of each sample was loaded onto a Novex NuPAGE (4–12%) Bis-Tris Mini Gel (Invitrogen, Carlsbad, CA) in MOPS buffer. NuPAGE Antioxidant was added to the upper buffer chamber for reduced samples. After electrophoresis, test article banding patterns were compared to Novex Sharp Standards as a molecular weight reference.

All gels were fixed using a solution of 25% acetic acid, 10% propanol for at least 20 minutes before staining with 0.006% Brilliant Blue G-250 in 10% acetic acid overnight. Destaining was achieved using 10% acetic acid, replaced twice before imaging the gels.

## 3. Results and Discussion

### 3.1. Preliminary DoE Results

Our model IgG3 antibody has been established over time to present a stringent challenge model for formulation development. Its amino acid sequence (Genbank protein sequence ID's AKH40268 and AKH40269) establishes it as a murine IgG3:*κ* with V_*κ*_4 and V_H_1-S121 regions. To scout individual buffer species, the IgG3 antibody was formulated with variable NaCl concentration and pH ranges and evaluated for gross stability of the antibody. Single buffer species formulations were chosen based on historical formulation experience and known acceptable pH ranges. Many of these formulations were eliminated as candidates based on the* T*0 analytics that indicated decreased solubility and decreased stability of the antibody. Absorbance at 410 nm (a surrogate for opalescence) and SEC proved to be sensitive measurement of solubility and stability of the antibody. These data guided the 2^4^ full factorial DoE based on histidine/arginine buffer formulations as described below.

#### 3.1.1. Acetate

**Figure 1:**
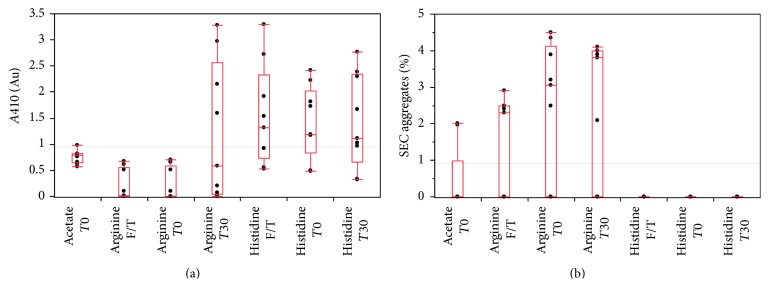
Quantile graphs of the buffer formulations at all measurement points. (a) Recorded absorbance of the samples at 410 nm and (b) the percent aggregate as determined by SEC. Histidine formulations showed gross precipitation so large that they are captured by a SEC-column frit during analysis and this may have led to a false negative of percent aggregates (see Section 3.1.2).* T*0 denotes initial time point,* T*30 denotes 30-day storage time point, and F/T denotes freeze/thaw.

**Figure 2:**
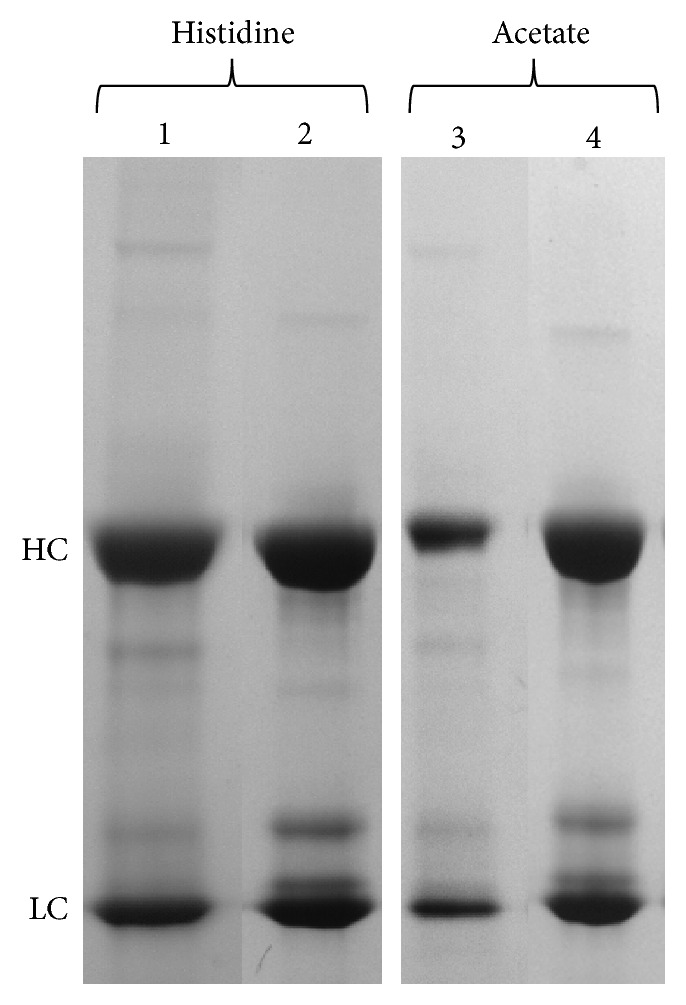
*Reduced SDS-PAGE. *HC denotes the heavy chain, while LC denotes the light chain of the antibody. Lanes 1 and 3 represent the insoluble fraction immediately after dialysis into the respective buffer system, while lanes 2 and 4 represent the supernatant.

All acetate buffer formulations showed visible precipitation during the small scale buffer exchange process. This observation was reflected in a high* A*410 reading coupled with a decreased* A*280. This unusual result indicated that the antibody was becoming insoluble as the acetate formulations replaced the 1 M arginine elution buffer during dialysis. This was verified in the SDS-PAGE showing heavy and light chain in the insoluble fraction of the buffer exchanged samples (Figure 2). All acetate formulations gave* A*410 readings greater than 0.5 (Figure 1(a), Table 3) and were therefore discontinued from further study. Although not a common lot release test employed by manufacturers,* A*410 acted as a measure for opalescence. This test quickly ruled out less desirable formulations by quantifying particulates. For our model antibody, insoluble aggregates in an abundance reflected in an* A*410 greater than 0.2 allowed us to focus our analytics on more promising buffer species. After this initial precipitation, the antibody maintained virtually 100% percent monomer, as measured by SEC, suggesting that components prone to nucleation precipitated completely, leaving behind monomer. The high percent monomer remaining was not beneficial enough to outweigh the solubility issues of acetate, therefore no further testing beyond a* T*0 time point was conducted on these formulations.

#### 3.1.2. Arginine

As expected, arginine improved solubility. At* T*0, arginine buffer formulations showed minimal opalescence, reflected in generally lower* A*410 values. The samples seemed to fall into two categories, moderate* A*410 around 0.5 and undetectable* A*410 (Figure 1(a)). The* A*280 remained stable after 30 days as well as after three freeze/thaw cycles proving that antibody did not grossly precipitate to the extent seen when formulated in acetate. Looking at all 9 formulations, there was decreased solubility at* T*30, as compared to* T*0, leading to minimal opalescence in some but not all formulations. These findings suggest that the arginine was conferring a cytoprotective effect, much like that seen when lyophilizing antibodies in arginine solutions [17]. The increased percent aggregates of the arginine buffer formulations as compared to acetate and histidine formulations (Figure 1(b), Table 3) arise from smaller aggregates that were not removed from the samples prior to running HPLC. Upon statistical analysis of the 9 formulations, we found that increased arginine concentration had the most overall positive effect on the antibody stability. We used this information to create an additional DoE to narrow our focus on higher concentration arginine in combination with a different buffering system at a more typical pH used for formulating antibodies.

#### 3.1.3. Histidine

Overall, the histidine buffer system showed even more extreme* A*410 versus acetate buffer at* T*0, which trended up by* T*30 as well as after the freeze/thaw procedure. This increase in opalescence over time was from the antibody becoming less soluble and forming large aggregates that completely fell out of solution, indicating that the antibody was increasingly unstable over time and after freeze/thaw cycles. These aggregates can be seen on the SDS-PAGE (Figure 2) and were removed before SEC analysis, leading to a misleading readout of 0% aggregate (Figure 1(b)). In addition, there was more variability in the* A*410 results, with the lower pH data points generally with lower opalescence (Table 3). Test formulations His 5 and His 6 both showed considerably lower absorbance at 410 nm as compared to the other buffers. This is likely due to the combination of high histidine (100 mM) and high salt (100 mM). Even after washing the insoluble fraction, the reduced SDS-PAGE of the histidine buffer formulations at* T*0 shows that there was a substantial amount of heavy and light chain in the insoluble fraction after buffer exchanging the antibody (Figure 2). These results indicated the particulates and precipitates formed were the drug substance and not host cell proteins or other insoluble components.* A*410 readings for histidine formulations were greater than 0.2 and discontinued from further study.

#### 3.1.4. Summary

Histidine and acetate as single buffer systems were eliminated in early rounds due to extensive opalescence in all DoE test articles (see Figures 1(a) and 2). Arginine, even at a pH close to the antibody isoelectric point, provided better results relative to the other two buffer systems, and stability correlated with higher arginine concentrations. This observation argues that instability was not a pH effect but that arginine was acting as a stabilizing agent. Thus, we further optimized the formulation buffer by retaining the presumed stabilizing effect of the arginine, while incorporating a second parameter that could provide buffering capacity at a pH (6.25 ± 0.25) sufficiently lower than the reported antibody isoelectric point (8.4–8.8) to help prevent self-association [15]. Histidine, even at lower concentrations, would provide this effect in combination with arginine. It was further noted that the stabilizing effect of NaCl was more pronounced when NaCl was at a higher concentration, across all three single buffer systems.

### 3.2. Second Round DoE

As described above in the single species buffer experiments, the antibody exhibited a modest trend towards better solubility at lower pH, and at higher arginine concentrations. We hypothesized that a combined histidine and arginine (His/Arg) DoE, at a pH further away from the antibody isoelectric point, could further minimize opalescence. In this case, histidine would buffer the pH below the pI of the antibody, while arginine would promote increased solubility and protein integrity due to chaotropic effects.

After statistical analysis of the* T*0 data, we found that there was a significant main effect for arginine buffer concentration. Lower arginine values (100 mM) were associated with higher levels of* A*410 absorbance, an undesirable indication for product quality. Additionally, while not statistically significant, but potentially biologically relevant, the arginine/histidine interaction (*P* = 0.05) and the histidine concentrations (*P* = 0.0547) are markedly more important than the remaining factors when considering strategies for minimizing* A*410 absorbance. Thus, by adjusting histidine concentration, we could design an optimal buffer to achieve the goal of low opalescence while also minimizing arginine addition, which could interfere in certain assays. The increased solubility achieved in the His/Arg DoE allowed us to select a final buffer formulation of 200 mM arginine, 50 mM histidine, and 100 mM NaCl at a pH of 6.5.

#### 3.2.1. Buffer DoE Freeze-Thaw and Stability

Bioprocessing usually occurs in separate drug substance and drug product facilities. This approach requires drug substance, and in some cases in-process material, to undergo freezing and thawing to allow shipping between distant sites. Regulatory agencies require specific studies that support hold times; these may include shipping studies of materials between facilities and long-term storage if not immediately processed into drug product [4]. While freeze/thaw is usually performed only once during shipping between drug substance and drug product sites, manufacturers may also study the impact of multiple freeze/thaws on product stability to understand risks posed by potential temperature deviations and unanticipated freezing and thawing. Poorly buffered formulations of other antibodies exposed to multiple freeze-thaw cycles have been shown to be prone to aggregation, subvisible particle formation that can ultimately nucleate visible aggregation [18]. This effect has been hypothesized to lead to undesirable product immunogenicity, although to an unknown degree [19]. They could also nucleate further aggregation during drug product fill operations [20]. Therefore, it is important to evaluate the drug substance stability over multiple freeze-thaw cycles and for extended hold times to evaluate the suitability of any buffer system.

**Figure 3:**
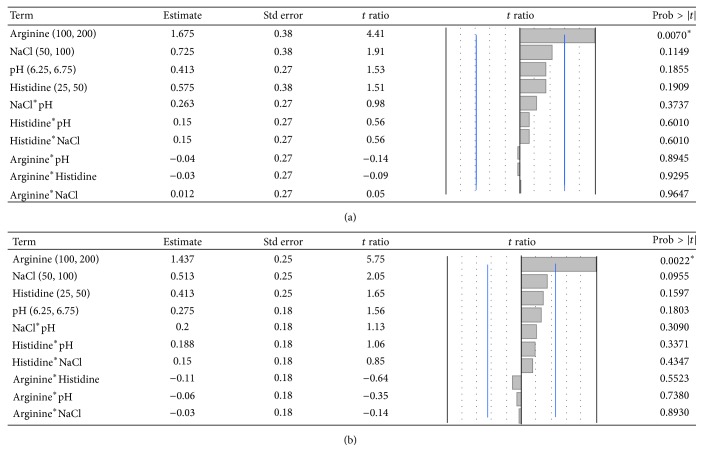
Significant effects on percent aggregate (a) shows that at* T*30, arginine concentration significantly reduced the aggregates. (b) After freeze/thaw, arginine played a significant role in reducing aggregation.

**Figure 4:**
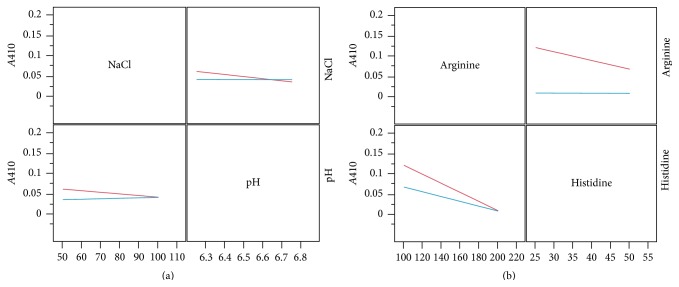
Significant interations on HR stability after both* T*30 (a) and freeze/thaw (b), and interactions between two variables lead to to significantly decreased* A*410. At* T*30 (a), NaCl concentration in combination with pH leads to a more desireable* A*410. After F/T (b), the interaction between arginine and histidine concentrations had a significant effect on* A*410.

**Figure 5:**
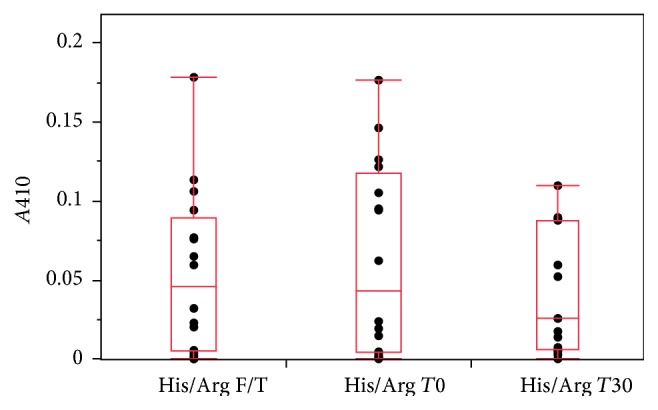
His/Arg A410 at each measurement point. Absorbance at 410 nm of 16 histidine/arginine buffer formulations as measured after the indicated time point.

To evaluate our His/Arg formulations for cryoprotection properties and extended hold times, we preformed the previously described analytics after 30 days of being held at 4°C, as well as three freeze/thaw cycles. Overall, we found that* A*410 was consistently more favorable among all 16 buffer formulations. The* A*410 of all the formulations from the combined DoE were below 0.2 AU (Figure 5) both over time and after freeze/thaw cycles. Not surprisingly, the significance of 200 mM arginine for reducing* A*410 values continued from the original* T*0, throughout the* T*30 and freeze-thaw studies. This was also reflected in a significantly decreased percent aggregates (Figure 3(a)). However, the importance of the arginine:histidine interaction became evident and statistically significant (*P* = 0.0476,* R* ^2^ = 0.97, *P* = 0.0355,* R* ^2^ = 0.96, resp.) (Figure 4). This value was well below the* A*410 achieved by the histidine formulations alone, and the 30-day stability in arginine formulations (Figure 1(a)).

We also evaluated antibody freeze/thaw stability. Upon three freeze-thaw cycles, arginine and the arginine-histidine interaction was statistically significant (*P* < 0.05,* R* ^2^ = 0.96) (Figures 3(b) and 4(b)). Histidine has previously been shown to reduce mAb aggregation in a concentration dependent manner under freeze/thaw conditions. Our results of an optimal histidine concentration of 50 mM coincide with observations from Chen et al. who found that 60 mM histidine showed a minimum amount of aggregates after 3 cycles of freeze/thawing [21]. It is often seen that when excipients are combined, the protective effects conferred on the antibody may not necessary increase [22]. The DoE format of our study allowed us to comprehensively evaluate the interactions of our chosen buffer species.

Overall, our observations indicate that the dual buffer system was improving the robustness and duration of the solubility of the antibody. An Arg:His interaction appears to allow for a lower arginine concentration if the other excipients are carefully balanced. The final buffer choice confers adequate solubility characteristics for short-term storage to allow additional studies of this antibody. This was important for other studies that depend upon its stability long enough to perform biochemical and physicochemical analysis.

## 4. Conclusions

As an individual component in a larger manufacturing process, bulk protein formulation choice is a critical step in antibody development. The right selection strategy choice can efficiently inform and assure that the best buffer choice will be made that enables drug product process robustness and ultimate product stability. An organized and directed approach can make the difference in determining if a biological candidate has a future for clinical or commercial use. Clearly, short-term, long-term, and freeze/thaw stability are critical considerations for this decision, as logistic constraints and shipping requirements are an inevitable part of the biotechnology manufacturing landscape. As we show here, even the stability of difficult to formulate antibodies can be vastly improved by careful, DoE-informed choice of buffering species and pH as well as controlled inclusion of stabilizing chaotropic agents. We also demonstrate that avoiding directly overlapping the antibody isoelectric point can minimize opalescence and precipitation.

## Highlights

- We used 4 DoEs to test 43 buffer formulations for stability of a model IgG3.
- Arginine increased the solubility of the model antibody.
- Combining 2 buffer systems, arginine and histidine, increased stability.
- Shifts in pH were a critical attribute affecting solubility of the antibody.

## Supplementary Material

The supplementary material provided gives the approach and data from the preliminary studies that guided the selection of levels and buffer DoE conditions described in the manuscript. Additionally provided is a detailed description of the composition of each buffer species that was considered in this study for the original 3 DoEs as well as the final, histidine/arginine DoE.

## Figures and Tables

**Table 1:** Single buffer DoE composition ranges. Levels for the individual buffer 2^3^ full factorial DoEs with center points.Each variable was assigned a high, middle, and low range before the full factorial was designed.

Buffer	Concentration (mM)	pH	NaCl (mM)
Acetate	25, 50, 100	4.5, 4.75, 5.0	25, 50, 100
Arginine	100, 200, 300	7.75, 8.0, 8.25	
Histidine	25, 50, 100	6.25, 6.5, 6.75	

**Table 2:** Full factorial DoE for dual buffer component (His/Arg) formulations. Detailed composition of each buffer tested in the 2^4^ full factorial DoE.

	Pattern	Arginine (mM)	Histidine (mM)	NaCl (mM)	pH
HR 1	++−+	200	50	50	6.5
HR 2	+−+−	200	25	100	6.0
HR 3	++−−	200	50	50	6.0
HR 4	++++	200	50	100	6.5
HR 5	−−−−	100	25	50	6.0
HR 6	−+++	100	50	100	6.5
HR 7	−+−+	100	50	50	6.5
HR 8	−++−	100	50	100	6.0
HR 9	−−+−	100	25	100	6.0
HR 10	−+−−	100	50	50	6.0
HR 11	−−−+	100	25	50	6.5
HR 12	+−++	200	25	100	6.5
HR 13	+−−+	200	25	50	6.5
HR 14	+++−	200	50	100	6.0
HR 15	−−++	100	25	100	6.5
HR 16	+−−−	200	25	50	6.0

**Table 3:** *T*0 analytic readout ranges for all DoEs for each buffer system; the range of values for A410, A280, and percent aggregates is given.This overview of the range of values gives a snapshot of how the different buffer systems compare to each other. ^*∗*^Gross precipitation of larger aggregates that would have been centrifuged out of solution before SEC or trapped by the column frit may have led to an artifactual 0% aggregate reading for antibody in the histidine formulations.

	Acetate	Arginine	Histidine	Histidine/arginine
*A*410	0.57–0.99	0–0.7	0.49–2.42	0–0.18
*A*280	2.11–3.7	2.23–2.93	2.22–8.88	1.36–2.24
Percent aggregates	0–2.0	0–4.5	0^*∗*^	0–3.87
