# Supplementary material for: Improved Stability of a Model IgG3 by DoE-Based Evaluation of Buffer Formulations
Source: Biomed Res Int. 2016 Mar 3;2016:2074149. doi: 10.1155/2016/2074149 (PMC4794585; doi:10.1155/2016/2074149)
Supplement: Supplementary file 1 — The supplementary material provided gives the approach and data from the preliminary studies that guided the selection of levels and buffer DoE conditions described in the manuscript. Additionally provided is a detailed description of the composition of each buffer species that was considered in this study for the original 3 DoEs as well as the final, histidine/arginine DoE. [file 2074149.f1.pdf]

	Concentration of Buffer	NaCl (mM)	pH	A410
Tris	100	100	8.0	0.587
	100	100	7.5	0.595
PO4	100	100	7.0	0.331
	100	100	6.5	0.928
Citrate	100	100	6.0	0.622
	100	100	5.5	0.369
Acetate	100	100	5.0	0.168
	100	100	4.5	0.116
Arginine	50	100	4.0	0.942
	50	100	8.0	0.214
Histidine	50	100	6.5	0.354

Supplemental Table 1: *Preliminary screening of buffer formulations.* Scouting data showing buffer composition and A410 readings taken immediately after overnight dialysis of the IgG3 antibody into the test buffer. The buffering species were chosen to cover a wide range of pH optima, while salt and substrate concentrations remained stable.

(A)

	Pattern	pH	Acetate (mM)	NaCl (mM)
Ace 1	−−+	4.5	25	25
Ace 2	+++	5	100	25
Ace 3	+−−	5	25	100
Ace 4	+−+	5	25	25
Ace 5	0	4.75	50	50
Ace 6	−++	4.5	100	25
Ace 7	−+−	4.5	100	100
Ace 8	++−	5	100	100
Ace 9	−−−	4.5	25	100

(B)

	Pattern	pH	Arginine (mM)	NaCl (mM)
Arg 1	−+−	7.75	300	25
Arg 2	−−−	7.75	100	25
Arg 3	+−+	8.25	100	100
Arg 4	+++	8.25	300	100
Arg 5	−−+	7.75	100	100
Arg 6	0	8.00	200	50
Arg 7	++−	8.25	300	25

Arg 8	−++	7.75	300	100
Arg 9	+−−	8.25	100	25

(C)

	Pattern	pH	Histidine (mM)	NaCl (mM)
His 1	++−	6.75	100	25
His 2	0	6.50	50	50
His 3	+−−	6.75	25	25
His 4	−−−	6.25	25	25
His 5	+++	6.75	100	100
His 6	−++	6.25	100	100
His 7	+−+	6.75	25	100
His 8	−+−	6.25	100	25
His 9	−−+	6.25	25	100

Supplemental Table 2 (A-C): *Names and descriptions of individual buffer system formulations.* (A) describes the names, patterns and formulations of each buffer tested in the Acetate system. (B) describes the same as panel (A), but for the Arginine system and (C) shows the Histidine system.
